# The Emerging Roles of Heterochromatin in Cell Migration

**DOI:** 10.3389/fcell.2020.00394

**Published:** 2020-05-27

**Authors:** Gabi Gerlitz

**Affiliations:** Department of Molecular Biology and Ariel Center for Applied Cancer Research, Faculty of Life Sciences, Ariel University, Ariel, Israel

**Keywords:** cell nucleus, chromatin, histones, genome organization, cancer metastasis

## Abstract

Cell migration is a key process in health and disease. In the last decade an increasing attention is given to chromatin organization in migrating cells. In various types of cells induction of migration leads to a global increase in heterochromatin levels. Heterochromatin is required for optimal cell migration capabilities, since various interventions with heterochromatin formation impeded the migration rate of numerous cell types. Heterochromatin supports the migration process by affecting both the mechanical properties of the nucleus as well as the genetic processes taking place within it. Increased heterochromatin levels elevate nuclear rigidity in a manner that allows faster cell migration in 3D environments. Condensed chromatin and a more rigid nucleus may increase nuclear durability to shear stress and prevent DNA damage during the migration process. In addition, heterochromatin reorganization in migrating cells is important for induction of migration-specific transcriptional plan together with inhibition of many other unnecessary transcriptional changes. Thus, chromatin organization appears to have a key role in the cellular migration process.

## Introduction

Chromatin is classically divided to euchromatin and heterochromatin. Euchromatin contains relatively open and active chromatin regions, while heterochromatin includes more condensed, gene-poor and less active chromatin regions (Carlberg and Molnár, 2019). Heterochromatin is subdivided to facultative and constitutive heterochromatin. The former contains repressed genes in a cell type-specific manner, while the latter is formed mainly over repetitive sequences and transposons localized at constant positions in various cell types such as pericentromeric regions, which are transcribed, although, at a very low level (Saksouk et al., 2015; Allshire and Madhani, 2018; Talbert and Henikoff, 2018; Marsano et al., 2019). Heterochromatin formation and maintenance is achieved by a battery of factors including histone variants, non-coding RNAs, DNA and histone modifications, factors that read these modifications, chromatin architectural proteins and chromatin remodeling factors (Allshire and Madhani, 2018). In general, DNA methylation is found in both types of heterochromatin, while facultative heterochromatin is enriched with the histone variant macroH2A, and the histone methylation marks H3K27me3, H2AK119Ub, and to less extent H4K20me1. Constitutive heterochromatin is enriched with H3K9me2/3 and H4K20me2/3 (Fodor et al., 2010; Fadloun et al., 2013; Mozzetta et al., 2015; Saksouk et al., 2015). These modifications promote chromatin condensation through the factors that bind them, which are termed readers (Soshnev et al., 2016). These readers include MeCP2, HP1 proteins, BAHD1 and L3MBTL1 (Canzio et al., 2014; Gozani and Shi, 2014; Mozzetta et al., 2015; Zhao et al., 2016; Tillotson and Bird, 2019). Increased nucleosome compaction in heterochromatin is achieved also by the chromatin architectural protein histone H1 that can be inhibited by phosphorylation (Hergeth and Schneider, 2015; Fyodorov et al., 2018) and chromatin remodeling factors such as ATRX (Clynes et al., 2013; Dyer et al., 2017). Not less important is the eviction of euchromatin markers out of heterochromatin regions (Allshire and Madhani, 2018). Historically, heterochromatin has been studied mainly in relation to regulation of gene expression during differentiation and development and to its supportive roles in cell cycle progression such as the importance of pericentromeric heterochromatin in cell division (Mozzetta et al., 2015; Saksouk et al., 2015; Allshire and Madhani, 2018; Talbert and Henikoff, 2018). However, in the last decade it has become apparent that heterochromatin levels are increased in response to cell migration signals and support better cell migration capabilities (Gerlitz and Bustin, 2011).

In animals, cell migration is a fundamental process in embryogenesis as well as in normal function of various tissues and systems such as regeneration of colon epithelium and the activity of the immune response. Mutations and deregulation of cellular migration processes are linked to various human diseases varied from intellectual disability to cancer metastasis (Nourshargh and Alon, 2014; Jiang and Nardelli, 2016; Reiner et al., 2016; Scarpa and Mayor, 2016; Stouffer et al., 2016; Lambert et al., 2017; Worbs et al., 2017; Chitty et al., 2018; Schumacher, 2019). In recent years it has been appreciated that the cell nucleus, which is the largest and most rigid cellular organelle has to undergo major changes in its position, structure and morphology during cell migration (Wolf et al., 2007, 2013; Friedl and Wolf, 2009; McGregor et al., 2016; Lele et al., 2018; Yamada and Sixt, 2019). Recent reviews covered thoroughly the emerging research field of the cell nucleus during migration while concentrating on the nuclear envelope and its interactions with the cytoskeleton (Krause and Wolf, 2015; Bone and Starr, 2016; Liu et al., 2016; McGregor et al., 2016; Madrazo et al., 2017; Calero-Cuenca et al., 2018; Kengaku, 2018; Lele et al., 2018; Manley et al., 2018; Salvermoser et al., 2018). Here I focused on the major inner nuclear component, chromatin and more specifically on heterochromatin changes and their roles in cell migration.

## Heterochromatin Alterations in Migrating Cells

Induction of cell migration was found to trigger global chromatin reorganization in several cell types. Initial comprehensive analysis of global chromatin organization in migrating cells was carried out in mouse melanoma cells. In these cells, induction of migration in the wound healing assay led to a rapid increase in various heterochromatin markers that could be detected already 15–60 min after introducing the migration signals. These markers included the histone modifications H3K9me3, H3K27me3, and H4K20me1, a non-phosphorylated form of histone H1 and DNA methylation (Gerlitz et al., 2007; Gerlitz and Bustin, 2010; Maizels et al., 2017). In addition, chromatin residence time of the chromatin architectural proteins HMGA1 and HMGN2 that are involved in chromatin de-compaction turned shorter, while the chromatin residence time of histone H1 that increases chromatin compaction was prolonged (Gerlitz et al., 2007; Gerlitz and Bustin, 2010). In parallel, migrating cells were found to be more resistant to DNase I treatment compared to non-migrating cells, indicating an elevation in chromatin condensation levels in migrating cells (Gerlitz and Bustin, 2010).

Increased global chromatin condensation in response to migration signals was found in additional cell types: In human breast cancer cells H3K9me3 levels were increased in response to expression of the activated form of Amphiregulin (AR). AR is an EGF family member that upon activation undergoes cleavage and translocation from the plasma membrane to the inner nuclear membrane while activating cell migration (Isokane et al., 2008; Tanaka et al., 2012). In human and mouse CD4^+^ T-cells induction of migration by Vascular Cell Adhesion Molecule 1 (VCAM1) led to an increase in H3K9me2/3 levels and to a higher resistance of the genome to cleavage by DNase I and MNase (Zhang X. et al., 2016). In rat tenocytes increased DNA methylation levels and genome resistance to DNase I cleavage were identified upon induction of migration by mechano-growth factor E peptide (MGF-C25E) (Zhang B. et al., 2016). In bone marrow-derived mesenchymal stem cells higher H3K27me3 levels and increased resistance to DNase I cleavage were found after induction of migration by the chemokine-like extracellular matrix (ECM)-associated protein Osteopontin (Liu et al., 2018). *In vivo*, DNA methyltransferases (DNMTs) levels and DNA methylation levels were shown to increase during wound healing of mouse corneal epithelium (Luo et al., 2019) and in colorectal cancer, H3K9me3 levels were found to be higher in the tumor invasive front than in non-invasive parts of it (Yokoyama et al., 2013). Interestingly, heterochromatin reorganization in response to migration signals was found not only in mammalian cells, but also in the filamentous fungus *Neurospora crassa*, in which accumulation of histone H1 was detected in the leading edge of migrating nuclei (Freitag et al., 2004; Gerlitz et al., 2007), thus heterochromatin reorganization in migrating cells may be an evolutionary conserved feature.

Recently, chromatin in migrating cells has been analyzed using higher resolution next generation sequencing tools. Chromosome conformations were captured by the Hi-C technique in human neutrophil-like cells that migrated through large pores (14 μm in diameter) and through confined pores (5 μm in diameter). As anticipated, migration through narrower pores associated with a higher degree of changes in chromosome conformations. Interestingly, disruptions of short-range interactions and of topologically associating domains (TADs) occurred to a higher extent in heterochromatin regions (compartment B in Hi-C analysis) than in euchromatin (compartment A) (Jacobson et al., 2018). Detailed analysis of heterochromatin was carried out in migrating mouse melanoma cells by a ChIP-seq analysis of the heterochromatin markers H3K9me, H3K27me3, and H4K20me1. Interestingly, upon induction of migration these markers were found to spread over larger genomic regions, while accumulating to a lesser extent, in specific genomic loci to form peaks. Though smaller in number, the migration-specific peaks of H3K9me3 and H4K20me1 accumulated over repetitive regions, while the ones of H3K27me3 accumulated over genes (Segal et al., 2018). Thus, signatures of both facultative and constitutive heterochromatin have been found to be highly dynamic in migrating cells.

## Effects of Heterochromatin Levels on Cell Migration Rate

Indications that global chromatin condensation is important for cell migration emerged from numerous experiments in which interference with heterochromatin formation attenuated the migration rate of a vast variety of cells. Knockdown or chemical inhibition of EZH2, which is the catalytic subunit of the H3K27 methyltransferase complex PRC2, inhibited the migration rate of various cell types (Table 1). Interfering with H3K9me2/3 levels by knocking down methyltransferases that generate these modifications such as G9a, SUV39H1, SUV39H2, SETDB1, and SETDB2 or by using chemical inhibitors of G9a and SUV39H1/2 also inhibited the migration rate of many cell types (Table 1). On the other hand, over-expression of H3K9me2/3 methyltransferases was shown to enhance the rate of cell migration (Table 1).

**TABLE 1:** Studies identifying the dependence of cell migration on heterochromatin levels.

**Heterochromatin alteration and the effect on migration**	Cell type (*-primary cells)	**Method of manipulation**	**Migration assay**	**References**
H3K27me3 reduction leading to inhibition of migration	Bone marrow-derived mesenchymal stem cells*	EZH2 siRNA	TA	Liu et al., 2018
	Mouse embryonic fibroblasts*	EZH2 siRNA	TA and WH	Kottakis et al., 2011
	Endometriotic epithelial cells	EZH2 siRNA and inhibitor (GSK126)	TA and WH	Eskander et al., 2013; Zhang et al., 2017
	Squamous cell carcinoma	EZH2 siRNA and inhibitors (GSK126 and EPZ-6438)	WH	Adhikary et al., 2015
	Immortalized keratinocytes	EZH2 siRNA	WH	Adhikary et al., 2015
	Melanoma	EZH2 siRNA and inhibitors (GSK126 and GSK343)	TA and WH	Luo et al., 2013; Maizels et al., 2017
	Pancreatic cancer cells	EZH2 siRNA	TA and WH	Ma et al., 2018
	Ovarian cancer cells	EZH2 siRNA	TA	Lu et al., 2010; Rao et al., 2010
	Breast carcinoma cells	EZH2 siRNA	TA	Varambally et al., 2008
	Prostate cancer cells	EZH2 siRNA	WH	Varambally et al., 2008
H3K27me3 enhancement leading to acceleration of migration	Pancreatic cancer cells	EZH2 OE	TA and WH	Ma et al., 2018
H3K9me2/3 reduction leading to inhibition of migration	Vascular smooth muscle cells*	SUV39H1 siRNA	TA	Zhang et al., 2019a
	Melanoma cells	SUV39H1/2 inhibitor (chaetocin) and SETDB1 siRNA	TA and WH	Gerlitz and Bustin, 2010; Maizels et al., 2017; Orouji et al., 2019
	Liver cancer cells	SETDB1 siRNA	TA	Wong et al., 2016; Zhang et al., 2018
	Gastric cancer cells	SETDB1 siRNA	TA	Nishikawaji et al., 2016
	Lymphocytes	G9a siRNA and inhibitor (BIX01294)	TA and Collagen matrix assay	Zhang X. et al., 2016; Madrazo et al., 2018
	Glioma cells	SETDB1 and SUV39H1 siRNA, SUV39H1/2 inhibitor (chaetocin)	WH	Spyropoulou et al., 2014; Sepsa et al., 2015
	Breast cancer cells	SUV39H1 siRNA and SUV39H1/2 inhibitor (chaetocin)	TA and WH	Yokoyama et al., 2013
	Cervical cancer cells	G9a inhibitor (BIX01294)	TA and WH	Chen et al., 2017
	Lung cancer cells	G9a siRNA, inhibitor (BIX01294) and DN	TA and WH	Chen et al., 2010; Huang et al., 2017
	Colon cancer cells	SETDB1 siRNA	TA	Yu et al., 2019
	Melanoma cells	SUV39H1/2 inhibitor (chaetocin)	TA and WH	Gerlitz and Bustin, 2010; Maizels et al., 2017
H3K9me2/3 enhancement leading to acceleration of migration	Vascular smooth muscle cells*	SUV39H1 OE	TA	Zhang et al., 2019a
	Melanoma cells	SETDB1 OE	TA and WH	Orouji et al., 2019
	Liver cancer cells	SETDB1 OE	TA	Zhang et al., 2018
	Gastric cancer cells	SETDB1 OE	TA	Nishikawaji et al., 2016
	Breast cancer cells	SUV39H1 OE	TA	Yokoyama et al., 2013
	Lung cancer cells	G9a OE	TA	Chen et al., 2010
	Colon cancer cells	SETDB1 OE	TA	Yu et al., 2019
Reduction in DNA methylation leading to inhibition of migration	Cortical interneurons*	DNMT1 KO	Organotypic brain slice culture	Pensold et al., 2017
	Corneal epithelial cells*	DNMT1 siRNA	WH	Luo et al., 2019
	Breast cancer cells	DNMT inhibitors (AZA, SGI, C02S)	TA and WH	Shafiei et al., 2008; Su et al., 2018; Yuan et al., 2019
	Prostate cancer cells	DNMT inhibitor (AZA)	WH	Strmiska et al., 2019
	Ovarian cancer cells	DNMT inhibitor (AZA)	TA	Meng et al., 2013
	Lung cancer cells	DNMT1, 3a siRNA, DNMT inhibitor (AZA)	TA and WH	Mateen et al., 2013; Yan et al., 2014; Bu et al., 2018
	Glioma cells	DNMT3a,b siRNA, DNMT inhibitor (AZA)	TA and WH	Wang et al., 2015; Xu et al., 2015; Guo et al., 2017
	Esophageal cancer cells	DNMT inhibitor (AZA)	WH	Ahrens et al., 2015
	Osteosarcoma cells	DNMT inhibitor (AZA)	WH	Gong et al., 2019
	Pancreatic cancer cells	DNMT3b siRNA	TA and WH	Wang et al., 2019
	Colon cancer cells	DNMT inhibitor (AZA)	WH	Oshima et al., 2019
	Trophoblasts	DNMT inhibitor (AZA)	TA	Rahnama et al., 2006
DNA methylation enhancement leading to acceleration of migration	Lung cancer cells	DNMT3a OE	WH	Yan et al., 2014
	Liver cancer cells	DNMT3b OE	WH	Wu et al., 2019
Histone acetylation elevation leading to inhibition of migration	Bone marrow-derived mesenchymal stem cells*	HDAC inhibitor (TSA)	TA	Liu et al., 2018
	Neurons in *C. elegans* development*	HDAC1 mutations and HDAC inhibitor (TSA)	Whole animal development	Zinovyeva et al., 2006; Nambiar et al., 2007
	Schwann cells*	HDAC inhibitor (TSA)	TA	Wang et al., 2014
	Endothelial cells*	HDAC7 siRNA	WH	Mottet et al., 2007
	Smooth muscle cells*	HDAC4 siRNA and HDAC inhibitor (TSA)	TA	Yang et al., 2012; Usui et al., 2014
	Cardiac fibroblasts*	HDAC1 inhibition (ellagic acid)	TA	Lin et al., 2019
	Dendritic cells*	HDAC inhibitor (TSA)	TA	Kim et al., 2013
	Tenocytes*	HDAC inhibitor (TSA)	WH	Zhang B. et al., 2016
	Melanoma cells	HDAC inhibitor (TSA)	TA and WH	Gerlitz and Bustin, 2010
	Breast cancer cells	HDAC2, 5, 8 siRNA, HDAC inhibitors (MS275, SB939, LBH, Tub, C02S, PCI-34051, VPA)	TA and WH	Jeon and Lee, 2010; Zhang et al., 2012; Hsieh et al., 2016; Li et al., 2016; Su et al., 2018; Yuan et al., 2019
	Ovarian cancer cells	HDAC3, 4 siRNA, HDAC inhibitor (TSA)	TA	Hayashi et al., 2010; Ahn et al., 2012; Meng et al., 2013
	Lung cancer cells	HDAC inhibitor (Silibinin)	TA	Mateen et al., 2013
	Esophageal cancer cells	HDAC inhibitor (MS-275)	WH	Ahrens et al., 2015
	Transformed macrophages	HDAC inhibitor (Butyrate)	TA	Maa et al., 2010
	Oral cancer cells	HDAC2 siRNA	WH	Chang et al., 2011
	Prostate cancer cells	HDAC inhibitor (VPA)	TA	Wedel et al., 2011
	Glioma cells	HDAC3 siRNA	TA and WH	Zhu et al., 2013
Broad histone methylation inhibition leading to chromatin decondensation and inhibition of migration	Bone marrow-derived mesenchymal stem cells*	DZNep	TA	Liu et al., 2018
	Tenocytes*	MTA	WH	Zhang B. et al., 2016
	Chondrosarcoma	DZNep	WH	Girard et al., 2014
	Melanoma cells	MTA	TA and WH	Gerlitz and Bustin, 2010
Histone H1 alterations leading to inhibition of migration	Melanoma cells	OE of histone H1 DN	TA	Gerlitz et al., 2007
	Glioma, osteosarcoma and gastric cancer cells	OE of histone H1 DN	TA	Sang et al., 2019; Zhang et al., 2019b; Xu et al., 2020

*OE, over expression; DN, over expression of a dominant negative form; TA, transwell assay; WH, wound healing assay; SGI, Guadecitabine/SGI-110; MS275, Entinostat; Tub, Tubastatin A HCL; TSA, Trichostatin A; VPA, Valproic acid; DZNep, 3-Deazaneplanocin-A; MTA, 5′-deoxy-5′-methylthioadenosine.*

Inhibition of DNA methylation by 5′-aza-2′-deoxycytidine (AZA) or by knockdown of DNMTs also inhibited cell migration while over-expression of DNMTs was shown to enhance cell migration (Table 1). Interference with histone H1 chromatin binding by over-expression of a dominant form composed of histone H1 C’-terminal part or of phosphor-mimicking forms containing T to E mutations also altered cell migration rate (Table 1). Interference with chromatin condensation can be achieved also by increasing global histone acetylation through inhibition of nuclear histone deacetylases (HDACs) either by chemical inhibitors or by knockdown. As listed in Table 1 and in a recent review (Wawruszak et al., 2019), such manipulations also interfere with cell migration.

In most of the described cases the interventions with heterochromatin formation (e.g., introduction of siRNA or addition of a chemical inhibitor) were introduced ≥24 h before induction of migration. In such cases it is challenging to assess whether migration inhibition was due to failure of the cells to increase heterochromatin levels only upon receiving migration signals or due to alterations in their basal transcriptome. Changes in the basal transcriptome of non-migrating cells can turn it to a less favorable one for migration even before receiving any migration signals. This scenario is supported by the findings that the number of migration-altered genes and the degree of change at their expression levels are limited (Jacobson et al., 2018; Segal et al., 2018) as described below. Moreover, many of these experiments were done in cancer cells, which acquire a migration-supporting transcriptome already during the transformation process (Lamouille et al., 2014; Dhamija and Diederichs, 2016; Huang et al., 2019). Thus, in many cases it is hard to understand if basal heterochromatin levels or migration-induced heterochromatin levels are important for the migration process. Addressing this issue can be achieved by adding chemical inhibitors in parallel to the induction of migration as done only in few cases (Gerlitz and Bustin, 2010; Jeon and Lee, 2010; Wang et al., 2014; Huang et al., 2017; Maizels et al., 2017; Liu et al., 2018). In the future, this issue could be addressed by using degron-based systems (Röth et al., 2019) for rapid depletion of heterochromatin generating enzymes.

Notably, as described above, interference with signatures of both facultative and constitutive heterochromatin can interfere with cell migration rate suggesting that both types of heterochromatin can affect cellular properties important for the migration process.

## Heterochromatin Roles in Cell Migration

### Heterochromatin Mechanical Roles

Increased heterochromatin levels in migrating cells are spread over large genomic regions as could be detected by immunostaining of heterochromatin markers in various cells such as melanoma cells (Gerlitz et al., 2007; Gerlitz and Bustin, 2010; Maizels et al., 2017) as well as by high resolution mapping of these markers by ChIP-seq analysis in the same melanoma cells (Segal et al., 2018). This pattern supports global changes in the physical properties of the nucleus, since a global increase in heterochromatin levels induced by divalent cations was shown to elevate the stiffness of the nucleus in both isolated nuclei (Dahl et al., 2005) and nuclei in whole cells (Stephens et al., 2019). On the other hand, over-expression of HMGN5 or HMGA1, chromatin architectural proteins that oppose histone H1 chromatin binding and compaction, led to a reduction in nuclear stiffness (Furusawa et al., 2015; Senigagliesi et al., 2019). Chromatin decondensation by chemical inhibitors such as HDAC inhibitors and the methyltransferase inhibitor DZNep also found to reduce nuclear stiffness (Stephens et al., 2017, 2018; Liu et al., 2018; Krause et al., 2019). In agreement with this, atomic force microscopy (AFM) analysis of tenocytes and bone marrow-derived mesenchymal stem cells detected an increase in nuclear stiffness following induction of migration by chemokine-like agents (Zhang B. et al., 2016; Liu et al., 2018). Similar phenomenon was also reported in human and mouse CD4^+^ T lymphocytes upon activation of migration by VCAM1 (Zhang X. et al., 2016).

**FIGURE 1:**
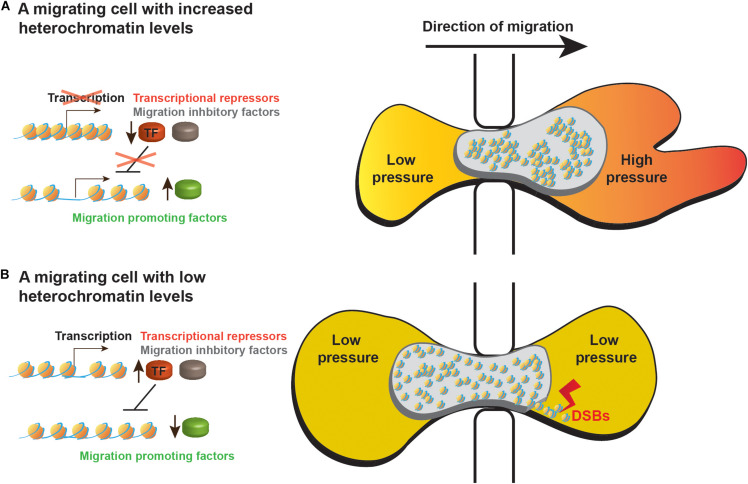
A model of heterochromatin roles in migrating cells. Schematic representation of cells migrating through small pores while **(A)** increasing their heterochromatin levels or **(B)** leaving their heterochromatin levels low as before receiving the migration signals. Higher heterochromatin levels support better the migration process by the following mechanisms: (i) Heterochromatin-dependent stiffness of the nucleus leads to faster nuclear movement out of the restraining pore. (ii) Increased nuclear stiffness may help the actomyosin network to increase the intracellular pressure in the anterior of the cytoplasm to induce formation of lobopodial protrusions. (iii) Increased nuclear stiffness may protect the nucleus of mechanical insults, preventing nuclear envelope rupture and DNA damage such as double strand breaks (DSBs). (iv) Heterochromatin inhibits transcription of migration inhibitory factors (marked in gray) and of repressors of transcription (TF, marked in red) thus preventing transcriptional inhibition of migration promoting factors (marked in green). (v) Heterochromatin also prevents unnecessary transcriptional alterations.

A first indication that indeed global heterochromatinization supports cell migration by altering the nuclear mechanical properties emerged of the finding that a HDAC inhibitor inhibited melanoma cell migration during a short period of time (3 h) in a similar efficiency also when transcription was inhibited (Gerlitz and Bustin, 2010). More recently, a detailed analysis of colon cancer migration through confined spaces revealed that heterochromatin-dependent nuclear stiffness generated a bigger forward jump of the nucleus once it is extracted from a narrow pore (Krause et al., 2019). Thus counter-intuitively, heterochromatin increased nuclear elasticity to generate a better spring-like behavior of the nucleus that can better support movement of the whole cell. An additional 3D migration mode that may benefit from altered nuclear physical properties by global heterochromatin formation is the nuclear piston model, which was identified in primary human cells and can be activated in tumor cells by inhibition of matrix metallopeptidases (MMPs). MMPs cleave the extra cellular matrix to facilitate easier migration of cells. 3D migration by the nuclear piston mechanism involves forward pulling of the nucleus by the actomyosin system in cooperation with the nucleoskeleton linker protein Nesprin 3. Due to the narrow diameter of a cell migrating inside the ECM, nuclear pulling divides the cytoplasm into two compartments. In the anterior compartment, the forward pulling of the nucleus by the actomyosin system increases the intracellular pressure. This pressure was found to promote formation of lobopodial protrusions that support forward movement of the cell (Petrie et al., 2014, 2017). Global heterochromatinization that increases nuclear stiffness may generate a nucleus that will not collapse and will deform only to the right degree that is required to compartmentalize the cytoplasm of a migrating cell (Figure 1). During 2D migration, we hypothesize that increased nuclear stiffness could improve momentum transfer of forces generated by the actomyosin network at the back of the nucleus leading to a more efficient usage of these forces to move the nucleus forward.

Higher nuclear stiffness in migrating cells might increase resistance to shear stress that can tear the nucleus. Recent studies on the cell nucleus during 3D migration showed that this process is associated with nuclear blebbing, nuclear envelope rupture and DNA damage that are inversely linked to the diameter of the pores thorough which cells migrate (Denais et al., 2016; Raab et al., 2016; Irianto et al., 2017; Pfeifer et al., 2018; Mistriotis et al., 2019). Notably, chromatin decondensation by chemical inhibition of HDACs or methyltransferases was shown to increase nuclear blebbing, while induction of chromatin condensation by treating cells with a histone demethylase inhibitor was found to reduce nuclear blebbing (Stephens et al., 2018, 2019). Thus, chromatin condensation during cell migration may increase the whole nucleus resistance to shear stress and reduces the susceptibility of DNA to breaks (Figure 1). This hypothesis is supported by the findings that applying mechanical stress on nuclei either by pulling them into small micropipettes or by exposing cells to a biaxial extrinsic cyclic mechanical strain led to global chromatin condensation (Irianto et al., 2016; Le et al., 2016).

### Heterochromatin in Transcriptional Control

One of the major roles of heterochromatin is considered to be repression of gene expression and transposons (Allshire and Madhani, 2018), however, a global reduction in transcription levels was found only in breast and ovarian cancer cells that were induced to migrate by an activated form of AR. This reduction was transient and prolonged for only 8 h (Isokane et al., 2008; Tanaka et al., 2012). In other cases such a repression was not identified (Fitsialos et al., 2007; Demuth et al., 2008; Jacobson et al., 2018; Segal et al., 2018). Moreover, active transcription is required for cell migration as the migration process continues for 8 h and more (Gerlitz and Bustin, 2010; Mason et al., 2019). Significantly, induction of migration is associated with specific changes in the cellular transcriptome in the scale of a few hundreds of genes (Fitsialos et al., 2007; Demuth et al., 2008; Jacobson et al., 2018; Segal et al., 2018). Using an EZH2-specific inhibitor to prevent H3K27 methylation upon induction of migration, in melanoma cells, revealed that H3K27 methylation is required for 33% of the 182 transcriptome changes in migrating cells. Surprisingly, H3K27 methylation was also found to prevent changes in 501 other genes that normally do not change upon induction of migration (Segal et al., 2018). Thus, migration-induced heterochromatinization is served not only to induce needed transcriptional changes, but also to prevent or to buffer unnecessary transcriptional changes. These unnecessary transcriptional changes may occur due to activation of transcription factors with multiple target genes of which only a fraction should be altered (Figure 1). A buffering role of heterochromatin in migrating cells could be seen also in migration of neutrophil-like cells, where interference with 3D genome structures occurred to a higher extent in heterochromatin regions than in euchromatin regions (Jacobson et al., 2018).

Overall, recent studies indicate that heterochromatin in migrating cells has physical roles in nuclear biomechanics as well as genetic roles in regulation of transcription. Although it is tempting to speculate that constitutive heterochromatin is important for the former roles, while facultative heterochromatin is important for the later roles, a complete analysis to support such a hypothesis has not been done yet. The findings that heterochromatin is used both to modify transcription and to prevent transcriptional changes suggest that altering the transcriptome of migrating cells should interfere with their migration rate. Especially, if the interference starts hours before induction of migration, thus it can alter the basal transcriptome. Indeed, there are studies in which interference with euchromatin markers also inhibits cell migration (Kim et al., 2018; Wang et al., 2018; Liang et al., 2019).

## Concluding Remarks

Cell migration is a key process in metastasis formation in cancer. Indeed, several heterochromatin generating enzymes such as the H3K9 methyltransferases G9a and SETDB1 and the H3K27 methyltransferase EZH2 are considered oncogenes (Tiffen et al., 2015; Kang, 2018; Batham et al., 2019; Cao et al., 2019; Torrano et al., 2019), whereas the H3K27 demethylases UTX and JMJD3 are considered tumor suppressor genes, though exceptions can be found (Arcipowski et al., 2016; Perrigue et al., 2016). Epigenetic drugs that interfere with heterochromatin formation such as DNMT inhibitors and HDAC inhibitors are used in cancer treatment (Castillo-Aguilera et al., 2017; Pechalrieu et al., 2017; Roberti et al., 2019). Unfortunately, a first-order link between heterochromatin and cancer does not always exist. In recent years it has become apparent that cancer cell proliferation and migration may be supported by different transcriptional plans (Nair et al., 2019; Luo et al., 2020) as well as by different global chromatin organization features; in melanoma it seems that euchromatin supports better cell proliferation, whereas increased chromatin condensation (heterochromatin levels) better supports cell migration (Barsotti et al., 2015; Maizels et al., 2017). Thus, targeting cancer cells by a single epigenetic drug might be challenging.

The opposing effects of heterochromatin on cell migration and proliferation suggest that if a heterochromatin marker is kept at the end of the migration process as an epigenetic memory, it may interfere with proliferation. Indeed, in migrating melanoma cells H3K27me3 levels were shown to drop back to basal levels once migration ended (Gerlitz and Bustin, 2010). Still, further studies are required to reveal if epigenetic memory of previous migration episodes can be formed to enhance future migration sessions in non-proliferating cells or in cancer cells in which the proliferation process is not sensitive to high heterochromatin levels as in melanoma cells.

Heterochromatin spatial organization inside the nucleus is not uniform; in most differentiated cells a substantial part of heterochromatin accumulates at the nuclear periphery next to the nuclear envelope (van Steensel and Belmont, 2017). However, in relation to migration it was only found that activation of migration of CD4^+^ T lymphocytes induced association of the H3K9 methyltransferase G9a with the nuclear envelope protein lamin B1 (Zhang X. et al., 2016). Thus, the spatial organization of heterochromatin in migrating cells is still unknown.

In recent years new links between heterochromatin and the nucleolus have been found. Pericentric heterochromatin is in close association with nucleoli while both structures use similar chromatin architectural proteins for their organization such as cohesion and HDACs (Bersaglieri and Santoro, 2019; Lawrimore and Bloom, 2019). Moreover, knockdown of the nucleolar protein STK35L1 was shown to reduce the migration rate of human endothelial cells (Goyal et al., 2011) and the histone acetyl transferase NAT10 was found to translocate from the nucleolus to the cytoplasm during colorectal transformation (Zhang et al., 2014). Thus, it is worthwhile to look for changes in nucleoli organization in migrating cells and for their roles in the migration process.

An additional important endeavor is to determine if heterochromatin formation upon induction of migration prevents DNA and nuclear damage during the migration process. To better understand the roles of heterochromatin in cell migration it is crucial to enlarge the pool of cell types and histone markers analyzed by next generation sequencing methods upon induction of migration in parallel to transcriptome analysis with and without interference with heterochromatin formation.

These suggested endeavors are important to further establish the emerging notion that chromatin in migrating cells is not a passive passenger, but rather an active player. Heterochromatin formation affects both nuclear mechanical properties and the transcriptome: heterochromatin adjusts the biomechanical properties of the nucleus for more efficient usage of force generated by the cytoskeleton as well as fine-tunes the cellular transcriptome while preventing changes that could impede cell migration rate.

## Author Contributions

GG screened the literature and wrote the manuscript.

## Conflict of Interest

The authors declare that the research was conducted in the absence of any commercial or financial relationships that could be construed as a potential conflict of interest.
